# Scabies Mite Peritrophins Are Potential Targets of Human Host Innate Immunity

**DOI:** 10.1371/journal.pntd.0001331

**Published:** 2011-09-27

**Authors:** Angela Mika, Priscilla Goh, Deborah C. Holt, Dave J. Kemp, Katja Fischer

**Affiliations:** 1 Queensland Institute of Medical Research and Australian Centre for International and Tropical Health and Nutrition, University of Queensland, Brisbane, Queensland, Australia; 2 Menzies School of Health Research, Charles Darwin University, Darwin, Northern Territory, Australia; National Institute of Allergy and Infectious Diseases, United States of America

## Abstract

**Background:**

Pruritic scabies lesions caused by *Sarcoptes scabiei* burrowing in the stratum corneum of human skin facilitate opportunistic bacterial infections. Emerging resistance to current therapeutics emphasizes the need to identify novel targets for protective intervention. We have characterized several protein families located in the mite gut as crucial factors for host-parasite interactions. Among these multiple proteins inhibit human complement, presumably to avoid complement-mediated damage of gut epithelial cells. Peritrophins are major components of the peritrophic matrix often found in the gut of arthropods. We hypothesized that a peritrophin, if abundant in the scabies mite gut, could be an activator of complement.

**Methodology/Principal Findings:**

A novel full length scabies mite peritrophin (SsPTP1) was identified in a cDNA library from scabies mites. The amino acid sequence revealed four putative chitin binding domains (CBD). Recombinant expression of one CBD of the highly repetitive SsPTP1 sequence as TSP-hexaHis-fusion protein resulted in soluble protein, which demonstrated chitin binding activity in affinity chromatography assays. Antibodies against a recombinant SsPTP1 fragment were used to immunohistochemically localize native SsPTP1 in the mite gut and in fecal pellets within the upper epidermis, co-localizing with serum components such as host IgG and complement. Enzymatic deglycosylation confirmed strong N- and O-glycosylation of the native peritrophin. Serum incubation followed by immunoblotting with a monoclonal antibody against mannan binding lectin (MBL), the recognition molecule of the lectin pathway of human complement activation, indicated that MBL may specifically bind to glycosylated SsPTP1.

**Conclusions/Significance:**

This study adds a new aspect to the accumulating evidence that complement plays a major role in scabies mite biology. It identifies a novel peritrophin localized in the mite gut as a potential target of the lectin pathway of the complement cascade. These initial findings indicate a novel role of scabies mite peritrophins in triggering a host innate immune response within the mite gut.

## Author Summary

The gut of most invertebrates is lined by a protective layer of chitin and glycoproteins, often designated as a peritrophic matrix. Previous research suggests that it forms a barrier that may protect the midgut epithelium from abrasive food particles and pathogens. Parasitic invertebrates ingesting vertebrate plasma have evolved additional strategies to protect themselves from hazardous host molecules consumed during feeding. An important part of the immediate defense in vertebrate plasma is complement-mediated killing. The Complement system is a complex network of more than 35 proteins present in human plasma that results in killing of foreign cells including the gut epithelial cells of a feeding parasite. Recently we found that scabies mites, who feed on skin containing plasma, produce several proteins that inhibit human complement within the mite gut. The mites excrete these molecules into the upper epidermis where they presumably also inhibit complement activity. Mite gut antigens that initially trigger the complement cascade have not been identified previously. Obvious possible targets of complement attack within the mite gut could be peritrophins. Our study describes the first peritrophin identified in scabies mites and indicates a possible role in complement activation.

## Introduction

Scabies is a widespread infectious parasitic disease [1]. The etiological agent, *Sarcoptes scabiei* burrows into the lower stratum corneum of the skin [2]. The clinical signs are erythematous lesions, pruritus and burrows [1]. Pruritus, commonly known as itchiness, is a consequence of a delayed type four hypersensitivity immune reaction [3]. Scabies is a major livestock disease [4] but animal scabies in humans is self limiting as the lifecycle of the mite cannot be completed.

Scabies spreads rapidly by person-to-person contact under crowded conditions. Indigenous Australians living in remote communities in the north of the country experience significant risk of morbidity from scabies. Pruritic scabies lesions facilitate opportunistic bacterial infections [5], particularly by Group A streptococci (GAS) and staphylococci [6]. According to a recent study undertaken in two communities, more than 70% of children presented to the health clinic with scabies by two years of age, with a peak of presentation at 2 months of age [6]. Importantly, in over 80% of these children skin sores were observed, indicating high rates of secondary infections with pathogenic bacteria. Among these particularly streptococcal infections cause significant sequelae (cellulitis, septicemia, and glomerulonephritis) and the increased community streptococcal burden has led to the most extreme levels in the world of Acute Rheumatic Fever and Rheumatic Heart Disease in these communities [5], [7].

Emerging resistance of scabies mites to current therapeutics emphasizes the need to identify novel targets for protective intervention [8]. Due to the difficulty of acquiring mites, no molecular studies on scabies were done until recently. Crusted scabies is a severe form of scabies with extreme parasite burden. We constructed cDNA libraries from mites obtained from skin shed in the bedding of patients with crusted scabies [9], [10], [11] facilitating molecular studies [12], [13], [14], [15]. A database containing over 43,000 expressed sequence tag (EST) sequences was created and searched for molecules predicted to play important roles in host parasite biology. One avenue pursued was to investigate gut proteins essential in food uptake. Gut molecules, being critical to parasite survival and concealed from the host's immune response but accessible for ingested antibodies, leucocytes and complement attack, are top-ranking targets in developing therapeutics against ticks and worms. For example the antigen Bm86 from the digestive tract of *Rhipicephalus microplus* (formerly *Boophilus microplus*) [16] was the basis for the development of the two anti-tick vaccines, which have been commercialized to date. The feeding scabies mite imbibes epidermal proteins and plasma containing host serine proteases, which mediate complement and blood clotting. Both systems must be inhibited in a milieu in which digestion of food can occur in the gut. Thus, in accordance with their parasitic life, scabies mites need to inhibit, control and prevent complement action in their gut.

The complement system of the vertebrate host forms a powerful immune barrier [17], [18], [19]. Upon entry of a foreign invader, this tightly regulated defense system is activated within seconds. Complement consists of approximately 35 proteins circulating in human plasma, penetrating mucosal surfaces and most tissues. The system targets foreign particles by attracting phagocytes to the site of damage, by increasing the permeability of the epidermal capillaries and by direct lysis of foreign cells. The complement system is triggered by various ligands and its activation can proceed via three enzymatic cascades: the classical, lectin or alternative pathways.

The classical pathway is initiated by the binding of the C1 complex to immune complexes, while the lectin pathway is activated via recognition of specific carbohydrates by mannose-binding lectin (MBL). The alternative pathway is triggered due to structural instability of the C3 molecule leading to autoactivation, direct binding of properdin or to an amplification loop to the two other pathways. Activation of any of these three pathways leads to the opsonization of the target with C3b, which mediates phagocytosis. Formation of C3b proceeds to the assembly of a membrane attack complex (MAC) and release of the chemoattractant anaphylatoxins C3a and C5a. The sequentially expanding pathways are vulnerable to inhibitors. Many pathogens utilize inhibitory molecules to block complement components and thereby evade host attack [20]. Among acarid arthropods tick salivary proteins have been shown to inhibit a number of complement components. For example the complement inhibitor OmCI, a salivary protein released by the soft tick *Ornithodoros moubata,* has been shown to directly bind to C5 in a stable complex 86 [21], thereby inhibiting cleavage of C5 into the anaphylatoxin C5a and the subunit of the membrane attack complex C5b. This inhibition protects the tick from complement-mediated damage by either the classical or alternative pathways. Similarly, *Ixodes scapularis* and *I. ricinus* produce a family of homologous Isac-like proteins that inhibit the alternative pathway by preventing the deposition of C3b and the release of C3a [22]. In addition one of these homologs, *I. scapularis* salivary protein 20 (Salp20), was shown to bind to the regulatory protein properdin, thereby destabilising the complement C3 convertase (C3bBb) in the alternative complement pathway [23].

Our group has previously identified a protein family of catalytically inactive scabies mite serine proteases (SMIPP-Ss [12]), which are secreted into the gut and excreted into the epidermis [12], [24]. They are homologous to the group 3 allergens of house dust mites but lack proteolytic activity due to mutations in the catalytic triad and structural rearrangements [24], [25]. All SMIPP-Ss examined to date were found to exert complement-inhibitory properties and two exemplary SMIPP-Ss have been shown to bind to the complement factors C1q, MBL and properdin [26]. The fact that a large multigene family has evolved to inhibit all three pathways of the complement cascade indicates that host complement is a major threat to the mites. They may have a complement evasion machinery in place, which could have an impact on their microenvironment similar to that induced by associated bacterial pathogens in the epidermal burrows [19]. Moreover as both mites and bacteria release these complement inhibitors into the skin, they may even supplement each other's defense. An obvious question is which structures within the gut initiate the complement cascade, thereby requiring the mite to produce elaborate complement-inhibiting mechanisms? Peritrophic matrix (PM) proteins could be prime candidates in this role. They are thought to be present in the midgut of most invertebrates, protecting it against the abrasive food bolus, ingested pathogens and toxins. However, most knowledge collected to date is restricted to insect peritrophins [27]. Peritrophin proteins are major components of the insect peritrophic matrix known for their low solubility [28]. Given its important role, the arthropod peritrophic matrix has been investigated to develop new pest management strategies (reviewed in [27]). Intriguingly the scabies mite cDNA database [8], [9] contained an abundant peritrophin-like sequence. Here we present initial evidence this scabies mite peritrophin may be involved in activating complement.

## Methods

### Ethics Statement

All animals were handled in strict accordance with good animal practice as defined by the Australian code of practice for the care and use of animals for scientific purposes, 7th Edition, 2004 – Web site: http://www.nhmrc.gov.au/publications/synopses/ea16syn.htm and the National Health & Medical Research Council's (NHMRC) Animal Code of Practice. Ethical approval for the production of polyclonal antibodies in mice was obtained from the Queensland Institute of Medical Research Animal Ethics Committee in compliance with the Code of practice, the NHMRC, and the Queensland government responsible authority [29].

Written informed consent was obtained from the crusted scabies patient for the collection of shed skin crusts, with the approval of the Human Research Ethics Committee of the Northern Territory Department of Health and Families and the Menzies School of Health Research.

### Cloning

BLAST searches of the scabies mite cDNA database (http://vbc.med.monash.edu.au/~yvan/Sarcoptes_scabiei/login.php) identified a peritrophin-like sequence. The sequence encoding full length SsPTP1 was amplified from a cDNA library from human scabies mites [9], [10] using the primers 5′-accggtcgacTTCATCAATGGTCATCTGCATCGAAAACG-3′ and 5′-accgctgcagTCAGAATAGTTTGAAATAATTCGAATCTTCTGTGGTGG-3′ (Sigma), which incorporated the restriction sites *SalI* and *PstI,* respectively (lower case), required for directional cloning into the pQE9 vector (Qiagen). The PCR product was digested at these sites, ligated into the pQE9 vector, transformed into *E. coli* BL21 cells (Stratagene). Transformants were confirmed by sequencing with BigDye 3.1 (Applied Biosystems) using vector specific primers. The sequence of the first chitin-binding domain (CBD1) of the SsPTP1 sequence was amplified from the pQE9 clone containing the complete SsPTP1 sequence using specific primers 5′-accgccatggAATTCATCAATGGTCATCTGCATCGAAAACG-3′ and 5′-accgctcgagTCGAGGTTTTTTTGTAGTATTTTTTCTGAACCA-3′ (Sigma) incorporating the restriction sites *Nco1* and *Xhol* and directionally cloned into a modified version of the pET-41a vector (Novagen) containing a tetraspanin protein 2 fragment from *Schistosoma mansoni* [30]. To obtain a peptide without a large N-terminal fusion for immunizations, fragment CBD1 was amplified using identical primers but with the restriction sites *SalI* and *PstI* and directionally cloned into the pQE9 vector (Qiagen). The CBD1 constructs were transformed into BL21 *E. coli* cells. Each of the three fusion constructs contained a C-terminal hexaHis-tag.

### Heterologous Expression and Purification

Recombinant CBD1 and TSP-CBD1 proteins were expressed and purified under denaturing conditions. *E. coli* cells were cultivated in LB medium containing 100 µg/mL ampicillin at 37°C over night. After inoculation of 2YT medium containing the same concentration of ampicillin, the cells were grown at 37°C until an OD_600_ of 0.5-0.6 was reached and overnight expression was induced by addition of 1 mM IPTG. Cells were collected by centrifugation at 4000 x g at 4°C for 20 min, resuspended in 50 mM Tris, pH 8.0, 100 mM NaCl, 10 mM EDTA, 1 mM PMSF and lysed in the presence of 250 µg/ml lysozyme and 10 µg/ml DNAse at room temperature under continuous rotation. All of the following purification steps were performed at 4°C. After sonication of the spheroplasts by a Sonifier 250 (Branson), inclusion bodies were washed four times using 50 mM Tris, pH 8.0, 100 mM NaCl, 10 mM EDTA, 0.5% (v/v) Triton X-100 and retrieved by centrifugation (16,000 x g for 20 min at 4°C), followed by solubilization in 6 M guanidine hydrochloride, 50 mM Tris, pH 7.8, 1 mM DTT for 60 min. Proteins were further purified by nickel immobilized metal affinity chromatography (Qiagen): Solubilized protein was diluted 1∶1 with 6 M urea, 100 mM NaH_2_PO_4_, 10 mM Tris, pH 8.0, 5 mM imidazole, 150 mM NaCl, 1% (v/v) glycerol, 1 mM DTT and bound over night to a pre-equilibrated Ni-NTA matrix (Qiagen) on a rotating shaker. The matrix was loaded into a column (PolyPrep, BioRad) and washed with 10 column volumes of 6 M urea, 100 mM NaH_2_PO_4_, 10 mM Tris, pH 6.3, 5 mM imidazole, 150 mM NaCl, 1% (v/v) glycerol, 1 mM dithiothreitol. Bound proteins were eluted using 4 column volumes of 6 M urea, 100 mM NaH_2_PO_4_, 10 mM Tris, pH 8.0, 250 mM imidazole, 150 mM NaCl, 1% (v/v) glycerol and 1 mM dithiothreitol. For refolding of recombinant TSP-CBD1, 1 ml of protein eluted from Ni-NTA affinity chromatography was added in portions of 100 µl at a time in 5 min intervals at 4°C to 500 ml of refolding buffer (50 mM Tris – HCl, pH 8.0, 50 mM NaCl, 300 mM arginine, 5 mM dithiothreitol) under continuous stirring. Refolded protein was concentrated using an Ultrasette Lab Tangential Flow Device (PALL Life Sciences) with a 3 kDa filter pore size followed by further concentration using centrifugal filter units (Amicon Ultra, Millipore). Protein concentrations were determined according to [31]. Molecular masses and purity were confirmed using SDS-PAGE analysis with Coomassie blue R-250 staining. The integrity and identity of CBD1 was confirmed by mass spectrophotometry analysis, *i.e*. MALDI-TOF, at the Monash Biomedical Proteomics Facility at Monash University, Clayton, Victoria.

### Localization of SsPTP1 and Complement by Immunohistochemistry

A custom-made peptide (DRFGYFSHEDCWRFVL; c2) was purchased from Mimotopes, Australia conjugated with keyhole limpet hemocyanin (KLM). Antibodies against the recombinant CBD1 protein, dialyzed over night into PBS at 4°C, and the custom-made peptide were raised in BalbC female mice. Doses of 50 µg of protein were injected, resuspended in PBS with Alum adjuvant (PIERCE, USA). Pre-bleeds were collected before the first immunization dose to serve as negative controls. The antibody titers and specificity were tested by ELISA and western blot analysis.

Tissue preparation of human mite-infested skin samples and the immunolocalization of SsPTP1were accomplished as previously outlined [24]. Serial sections of 4 µm thickness cut from paraffin embedded human skin tissue infested with scabies mites underwent an Antigen heat-retrieval procedure in a solution containing 1% (w/v) Revealit-Ag in 20 mM Tris (pH 7.5; ImmunoSolution) at 75°C for 15 min. The slides were cooled and washed with Milli Q water for 10 min followed by 15 min washes in PBS at pH 7.2. The tissue sections were blocked with 10% donkey serum in 1% (w/v) BSA in PBS. Endogenous peroxidase activity was blocked with 3% (v/v) H_2_O_2_ in blocking buffer. The sections were incubated overnight with either mouse polyclonal antibodies against peritrophin c2-conjugate (1∶200 dilution) or polyclonal antibodies against recombinant peritrophin CBD1 (1∶500 dilution). The primary antibodies were probed with the secondary antibody Dako-EnVision anti-mouse-HRP conjugate (DakoCytomation) for 30 min. As a positive control, adjacent sections were incubated with anti-human IgG-HRP polyclonal antibody (Abcam) in 1∶500 dilutions to identify the mite's gut. As a negative control, pre-immune serum was incubated on sections of the same series in either 1∶200 or 1∶500 dilutions. All slides were washed in PBS and the Vector NovaRed peroxidase substrate kit (Vector Laboratories) was used for staining according to the manufacturer's recommendations, followed by counterstaining with hematoxylin. Slides were scanned using a Scan Scope XT microscope (Aperio Technologies) at 40× magnification.

For the localization of complement components a variety of blocking and immunodetection compounds and conditions were tested. As primary antibodies a rabbit anti-human SC5b-9 neoantigen-specific antibody recognizing the human MAC complex *in situ* after complement activation and a goat anti-human C9-antibody recognizing non-complexed C9 (Complement Technology Inc., USA) were used. The antibody specificity and titers required were tested by an ELISA-based complement depositon assay as described previously [26]. Tissue preparation of human mite-infested skin samples and the immunolocalization were accomplished as outlined above. The tissue sections were subjected to blocking with casein (BioCare Medical, USA) for 5 min at room temperature followed by overnight incubation with 10% goat serum in 1% (w/v) BSA in PBS for SC5b-9 detection and with 10% donkey serum in PBS for C9 detection. Adjacent sections were incubated with either the SC5b-9 neoantigen-specific antibody at 1∶200, 1∶500, 1∶1000, 1∶2000, 1∶4000 and 1∶20000 dilutions) or the anti-human C9-specific antibody at a 1∶200 dilution for 1h. Endogenous peroxidase activity was blocked with 3% (v/v) H_2_O_2_ in blocking buffer. The primary antibodies were probed with MACH 2 Rabbit HRP-Polymer (BioCare Medical, USA) for detection of SC5b-9 neoantigen and with Goat HRP-Polymer Kit (BioCare Medical, USA) for C9 detection. As a positive control for gut localization adjacent sections were incubated with anti-human IgG-HRP polyclonal antibody (Abcam) in 1∶500 dilutions. As a negative control, naïve pre-immune rabbit or goat serum was incubated on sections of the same series in the same dilutions as the test sera. The Vector NovaRed peroxidase substrate kit (Vector Laboratories) was used for staining according to the manufacturer's recommendations, followed by counterstaining with hematoxylin. Slides were scanned using a Scan Scope XT microscope (Aperio Technologies) at 40x magnification.

### Chitin Binding Assay

Magnetic chitin beads (NEB) were equilibrated with chitin binding buffer (20 mM Tris, pH 8.0, 500 mM NaCl, 1 mM EDTA, 0.05% (v/v) Triton X-100). Proteins were incubated with 500 µl of chitin beads for 2 hours at 4°C with rotary shaking in a total volume of 3 ml. Wheat germ agglutinin (WGA) as positive, and tetraspanin protein 2 fragment from *Schistosoma mansoni* (TSP; [30] and BSA as negative controls were used, respectively. After binding, the beads were washed three times with 10 column volumes binding buffer. The proteins bound to the beads were eluted in 1.25 ml fractions using 8 M urea, followed by the addition of 5% SDS and then 10% SDS.

### Deglycosylation of Native SsPTP1

30–40 mg scabies mites picked from pig ears [32] were homogenized in 150 ul 50 mM Tris, pH 8.0, 100 mM NaCl, 1 mM EDTA in an Eppendorf tube with a plastic pestle on ice. Deglycosylation was performed under denaturing conditions using an Enzymatic Protein Deglycosylation Kit (EDEGLY, Sigma) according to the manufacturer's instructions. PNGase F, O-glycosidase, α-neuraminidase, β-1, 4-galactosidase and β-N-acetylglucosaminidase were assayed separately and in combination. Enzyme reactions were incubated between 24 hours and three days at 37°C in a water bath and subsequently analyzed by SDS-PAGE.

### Western Blotting Using an Infrared Fluorescence Detection System

Proteins separated by SDS-PAGE were transferred onto an Immubilon-FL PVDF membrane (Millipore), blocked with Odyssey blocking buffer (*LI-COR* Biosciences) overnight at 4°C, and incubated for 1 h with 1∶500 dilutions of primary antibody raised against recombinant CBD1 protein. Membranes were washed four times in PBS, followed by incubation for 1h with a fluorescent secondary antibody (Goat anti mouse-IR dye λ_800 nm_) at 1∶10,000 dilution. Proteins were visualized using an Odyssey Infrared Imaging System (*LI-COR* Biosciences).

### Immunodetection of Human MBL Binding to Protein Mite Extract Using an Infrared Fluorescence Detection System

Fresh and deglycosylated mite homogenates were separated on SDS-PAGE and transferred onto an Immubilon-FL PVDF (Millipore) membrane. Membranes were incubated with 50% (v/v) normal human serum diluted in binding buffer, consisting of 50% GVB (2.5 mM veronal buffer, pH 7.3, 0.1% (w/v) gelatin) and 50% TBS (200 mM NaCl, 50 mM Tris pH 7.4), for 1 h at 37°C under continuous shaking. The membranes were washed four times in TBS, followed by 1 h incubation at room temperature with an anti-MBL antibody (AF2307, R&D Systems, Denmark) at a 1∶1000 dilution. The membranes were washed four times in PBS, followed by incubation with a fluorescent secondary antibody (Goat anti mouse-IR dye λ_800 nm_) at 1∶10,000 dilution. Protein detected by the antibody was visualized using an Odyssey Infrared Imaging System (*LI-COR* Biosciences).

### Software

Sequence analysis was performed using prediction tools available on www.expasy.org [33], [34], [35], [36]. Statistical analysis was done using Graph Pad Prism (GraphPad Software, USA).

## Results

### Sequence Analysis and Cloning of a Novel Scabies Mite Peritrophin

**Figure 1:**
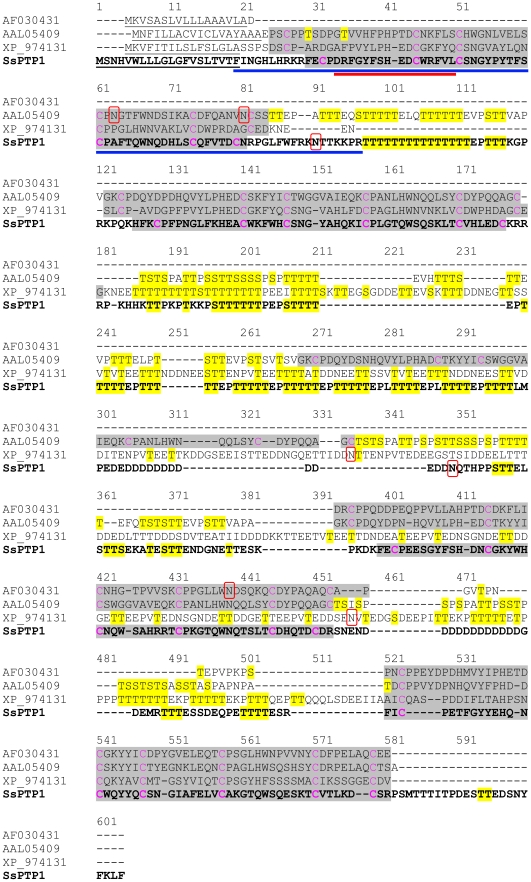
Full length amino acid sequence of scabies mite peritrophin 1 (SsPTP1) aligned with other arthropod peritrophins. The scabies mite peritrophin SsPTP1 is shown in bold in a sequence alignment with *Anopheles gambiae* peritrophin 1 (AF030431), peritrophin from *Aedes agypti* (AAL05409) and a peritrophin matrix protein from *Tribolium castaneum* (XP_974131). The following predictions are shown: signal sequences (underlined); chitin binding domains (grey background); N-glycosylated residues (red box); O-glycosylated residues (yellow background). Cysteine residues predicted to form disulfide bonds within the chitin binding domains are shown in magenta. A blue and a red bar indicate the sequence of the chitin binding domain CBD1 and the peptide c2, respectively.

BLAST searches of the scabies mite cDNA database identified a peritrophin-like sequence. The 1835bp contig of 18 ESTs (Genbank Accession number JF266566) included 1461bp of full length coding sequence. Based on a considerable amino acid similarity of 37-39% with previously reported arthropod peritrophins, the novel gene was termed *Sarcoptes scabiei* peritrophin 1 (*SsPTP1*). SsPTP1 had a predicted signal sequence of 20 amino acids, indicating that SsPTP1 might be a secretory protein. No transmembrane domains were predicted. Four chitin binding domains (CBDs) were detected each containing conserved patterns of six cysteines residues. The sequence showed a substantial degree of low complexity and repetitiveness including two threonine-rich domains between the CBDs (regions 97–111 and 255–298; Fig. 1). In addition, two N-glycosylation sites were predicted in SsPTP1 at position 90 and 283 of the amino acid sequence, while two regions with putative strong O-glycosylation were found (amino acids 97–116 and 185–312) indicated by the presence of repetitive serine and threonine residues in these regions (Fig. 1). The three aligned insect peritrophins were also analyzed for N- and O-glycosylation. The prediction showed that all four proteins may be hyper-glycosylated over the full length of their sequences with the exception of their chitin binding domains (Fig. 1).

*E. coli* expression and purification of full length recombinant SsPTP1 was without success due to the high repetitiveness of the sequence. The first chitin binding domain of SsPTP1 (CBD1) expressed in *E. coli* was insoluble, but could be purified in large amounts under denaturing conditions. Dialyzed and resuspended CBD1 was used to raise polyclonal antibodies in mice. In order to produce soluble proteins, CBD1 was additionally fused to the C-terminus of the schistosomiasis vaccine antigen *Sm*-TSP-2. Both proteins contained a C-terminal hexaHis tag. The fusion protein TSP-CBD1 was soluble after purification by nickel affinity chromatography from washed inclusion bodies and refolding, presumably because both fusion partners are highly soluble when expressed in *E. coli* [30].

**Figure 2:**
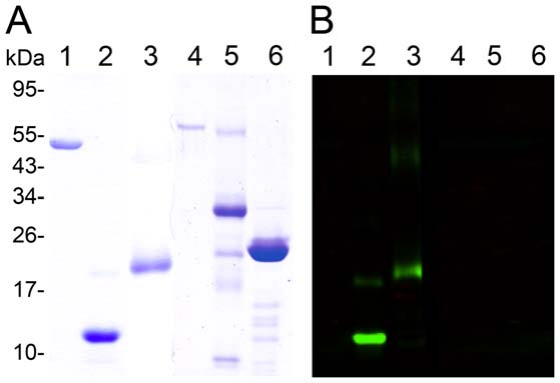
Specificity of mouse antisera raised against a recombinant chitin binding domain of SsPTP1. Shown is a series of purified scabies mite proteins on a coomassie-stained SDS-PAGE (A) and a corresponding western with a peritrophin-specific antibody (B). The gel (A) demonstrates the purity of the recombinant peptide CBD1 (lane 2) and the fusion protein TSP-CBD1 (lane 3) and (B) the specificity of the antibody raised against CBD1, confirming no cross-reaction with unrelated scabies mite proteins SMSB3a (lane 1), SMIPP-S D1 (lane 5), SMIPP-S I1 (lane 6) and BSA (lane 4).

CBD1 and TSP-CBD1 had the molecular masses of 12 kDa and 23 kDa, respectively (Fig. 2). After Ni-NTA purification, average protein yields of 73.9 ± 10.0 mg of TSP-CBD1 per 1 L *E. coli* culture were achieved. After optimization of the refolding procedure, up to 15.1% refolded protein was obtained. The soluble fusion protein was used in a chitin binding assay.

### A Recombinant SsPTP1 Chitin Binding Domain Binds Chitin in an Affinity Chromatography Assay

**Figure 3:**
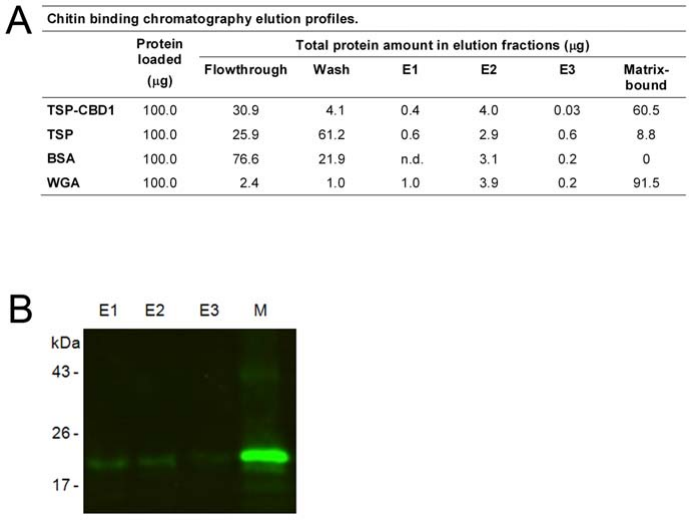
Binding of recombinant peritrophin fusion protein TSP-CBD1 by affinity chromatography to chitin magnetic beads. (**A**) Shown are elution profiles of the peritrophin fusion protein TSP-CBD1, *Schistosoma mansoni* TSP protein and bovine serum albumin (BSA) (negative controls), and wheat germ agglutinin (WGA; positive control). Data are means of n = 4 independent experiments measured in duplicate. 100 µg protein was loaded in each chromatography run. E1, elution (8M urea); E2, elution (5% SDS); E3, elution (10% SDS); matrix-bound, the amount of protein estimated to be still bound to the matrix after E1-E3 elution; n.d., not detectable (**B**) Western blotting of chitin binding chromatography fractions from an representative TSP-CBD1 run. Immunodetection was performed using polyclonal mouse antibodies against peritrophin peptide CBD1. Lanes E1-E3 show elution fractions and lane M protein bound to chitin matrix beads.

A substantial proportion of the fusion protein TSP-CBD1 bound to chitin magnetic beads. In comparison the chitin binding chromatography elution profiles of the purified tetraspanin protein 2 fragment from *Schistosoma mansoni* TSP and BSA as negative controls revealed small amounts of non-specific binding while wheat germ agglutinin (WGA) as a positive control showed strong binding (Fig. 3A). The total protein amounts found in all fractions including flowthrough and wash fractions showed significant differences suggesting that considerable amounts of TSP-CBD1 and WGA could not be removed from the matrix during elution. This was confirmed by Western blotting (Fig. 3B). Additional boiling of the chitin matrix in 10% SDS in elution buffer did not elute more protein (data not shown).

### Localization of SsPTP1 to the Mite Digestive System and Fecal Pellets in Human Skin

Polyclonal antibodies were raised in mice against a 16 amino acid custom-made peptide conjugated to keyhole limpet hemocyanine (c2). The specificity of both antisera to CBD1 and TSP-CBD1 was confirmed and no cross-reactions with unrelated scabies mite proteins or BSA were seen in western blot analysis. Representative data for anti-CBD1 serum from one mouse are shown in Fig. 2.

**Figure 4:**
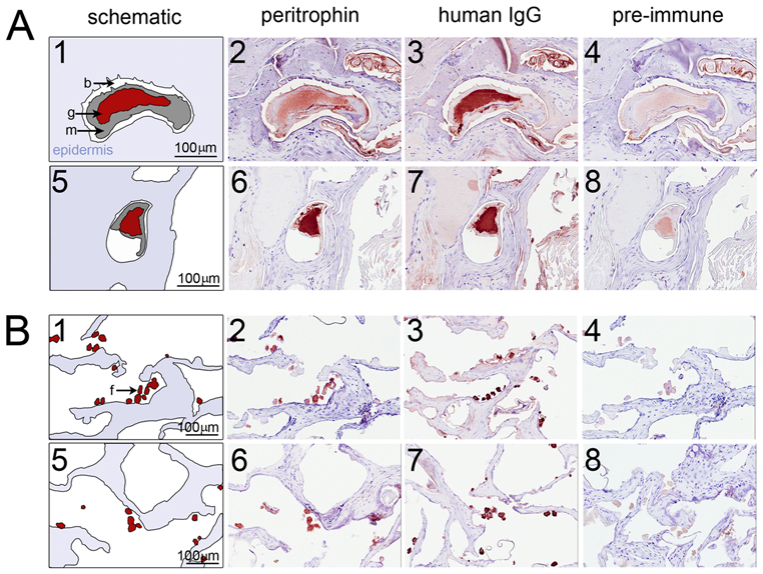
Immunohistological localisation of SsPTP1 in scabies mite gut and excreted mite feces. Histological sections were probed with antibodies raised against the peritrophin peptide CBD1 (A2, B2), against peptide c2 (A6, B6), with anti-human IgG (A3, A7, B3, B7) and corresponding pre-immune sera (A4, A8, B4, B8). Red staining indicates binding of primary antibody. A schematic diagram in the left panel (A1 and A5; B1 and B5) outlines the features in the histological sections. The mite gut and fecal pellets are shown in red, the mite body in gray, the burrow in white and the epidermis in blue. b, burrow; f, feces; g, gut; m, mite. Scale bars (100 µM) indicate the magnification level.

The SsPTP1 protein was localized within and outside the scabies mite using the polyclonal antibodies for immunohistochemical staining of serial sections of human skin infested with mites (Fig. 4). Each anti-SsPTP1 antibody showed strong staining of the digestive system of the mite (Fig. 4A- 2 and 6); while adjacent sections probed with pre-immune serum showed only minor background staining (Fig. 4A- 4 and 8). The immunodetection of human IgG, which was used as a positive control, confirmed the localization of the mite gut (Fig. 4A- 3 and 7) as previously documented [24], [37]. In addition to its location in the digestive tract of the mite, SsPTP1 was localized to external acellular masses, which are scybala (fecal pellets; Fig. 4B- 2 and 6). The scybala were negative for staining with pre-immune serum (Fig. 4B- 4 and 8) and positive for IgG staining. (Fig. 4B- 3 and 7). Given its presence in the digestive tract of the scabies mite and in excreted fecal matter, the peritrophin is likely to be involved in gut functions and would be predicted to be exposed to epidermal host proteins within the gut and externally in the epidermal burrow.

### Deglycosylation of Native Scabies Mite Peritrophin 1 and Binding to the Human Complement Factor MBL

**Figure 5:**
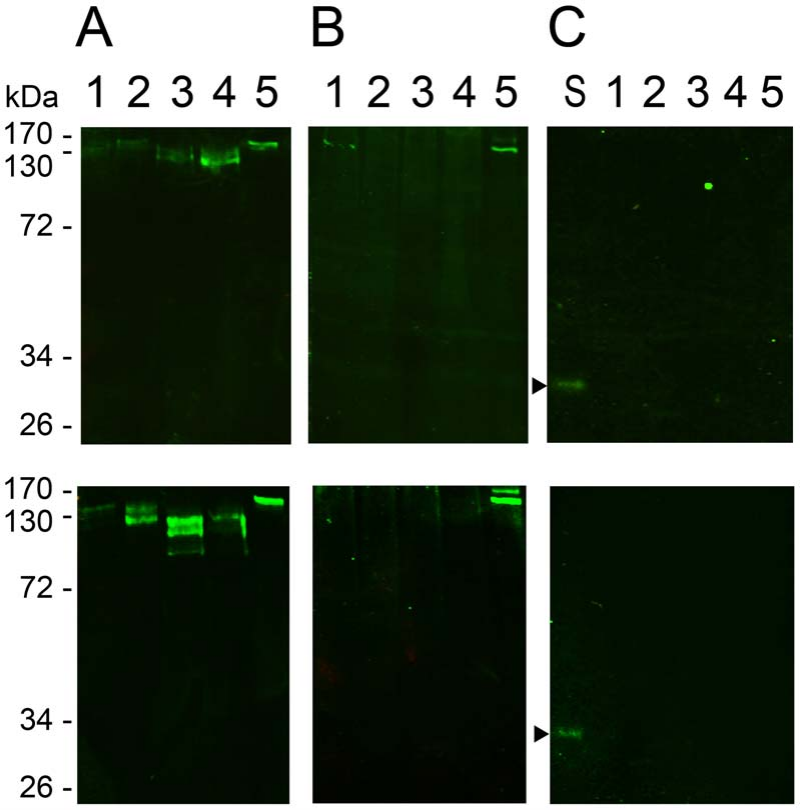
Deglycosylation of native scabies mite peritrophin 1 and binding to the human complement factor MBL. Scabies mite homogenates were incubated for 24 hours (upper panel) or 3 days (bottom panel) in either buffer alone (lane 1), or with PNGase F (lane 2), four O-glycosidases (lane 3), or PNGase F and four O-glycosidases (lane 4) at 37°C. The preparations were separated by SDS-PAGE in comparison with a sample of fresh mite homogenate (lane 5). Western blots were probed for SsPTP1 using anti-CBD1 sera (A), or for MBL using an anti-MBL antibody after incubation of the blot with 50% normal human serum (B) or with buffer without serum (C). In blot (C) an additional lane was loaded with 1% normal human serum in PBS (lane S). Arrows point to a single band corresponding to the 32kDa subunits of human MBL. The experiment was repeated three times.

Fresh scabies mite homogenates were subjected to deglycosylating enzymes for one (upper panel, Fig. 5) or three days (bottom panel, Fig. 5) at 37°C to remove N- and O-glycosylation of the proteins and subsequently separated by SDS-PAGE. A single band was detected by the anti-CBD1 antibody in untreated homogenates after Western blotting (Fig. 5A). Incubation of mite extract with glycosidases revealed smaller bands indicating enzymatic digestion of glycans and thus a strong glycosylation of the detected native protein (Fig. 5A). The material subjected to O-glycosidases showed the strongest decrease in molecular mass, especially after three days. In comparison PNGase F treatment showed a smaller effect. Hence in accordance with the large number of predicted O-glycosylation sites in SsPTP1 (Fig. 1), the O-glycosylation seemed to be predominant. Further membranes were incubated with 50% normal human serum containing all complement factors including MBL (Fig. 5B) or buffer without serum (Fig. 5C) followed by immunodetection of bound MBL using an anti-MBL antibody. Human MBL bound to a protein with a molecular mass corresponding to the band detected by the CBD1 antibody in the untreated mite extracts (Fig. 5B). Negative control experiments, lacking the incubation step in human serum (Fig. 5C) confirmed that i) the MBL antibody was specific for MBL. A distinct band (indicated by an arrow in Fig. 5C) corresponding to the 32 kDa-peptide polypeptide chains of the human MBL subunits [29] was detected. Furthermore this blot confirmed that ii) the MBL antibody did not detect putatively existing scabies mite haemolymph complement factors and iii) MBL was not detectable in the mite extract due to food intake using this method. Deglycosylation of the mite homogenate for one and three days revealed comparable results, however, the degree of visible deglycosylation was less after one day and protein degradation was more after 3 days. The results of the 24h digest suggest that the binding of human MBL to the detected protein may be highly dependent on the presence of carbohydrates.

### Immunohistological Detection of Complement Components in the Mite Digestive System

**Figure 6:**
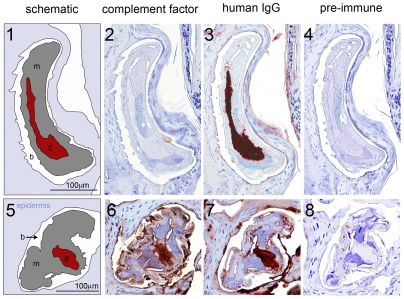
Immunohistological localisation detects complement factor C9, but not the activated SC5b-9 complex in scabies mite infested human skin sections. Histological sections were probed with antibodies raised against human SC5b-9 neoantigen (2) and against complement factor C9 (6), with anti-human IgG (3 and 7) and the respective pre-immune sera from goat (4) and rabbit (8). Red staining indicates binding of primary antibody. A schematic diagram in the left panel (1 and 5) outlines the features in the histological sections. The mite gut and fecal pellets are shown in red, the mite body in gray, the burrow in white and the epidermis in blue. b, burrow; f, feces; g, gut; m, mite. Scale bars (100 µM) indicate the magnification level.

Neoepitope-specific antibodies are suitable to discriminate between complement activation products and native complement factors. To detect whether *in situ* complement activation occurs in the mite gut we applied immunohistochemistry on scabies mites in human tissue using a neoepitope-specific antibody that recognizes the terminal surface-bound C5b-9 complex (MAC) after complement activation. Localization of C9 was detected using the same methodology. The antibody specificity and titers required were determined by an ELISA-based complement deposition assay (data not shown). While the presence of C9 was localised within the mite gut and in the epidermal tissue, no MAC formation was detected in the mite gut using this technique (Fig. 6).

## Discussion

Peritrophins have common structural elements which were similarly exhibited by SsPTP1 (Fig. 1). They are secretory proteins containing a signal peptide and between one and nineteen CBDs [38], [39], [40], [41], [42]. Cleavage sites for serine proteases are often located downstream of these domains, and it was postulated that processing into smaller units occurs, when the molecules are integrated into the PM [43]. As another common motif, the CBDs contain conserved patterns of six, eight or ten cysteine residues which form intra- and inter-molecular disulfide bridges that are important for the interaction with chitin microfibrils.

Four CBDs were identified in SsPTP1, each containing six conserved cysteines which may form three intra-molecular disulfide bonds, possibly facilitating a tertiary structure that may assist in binding of aromatic residues to GlcNAc residues in the chitin fiber as suggested for other peritrophins [28], [40]. This arrangement is thought to be the key element to the strength and structure of the PM [44], [45]. Disulfide bonds in peritrophin-44 for example were reported to confer a substantial level of resistance to protein degradation [41]. Resilience to proteolysis is essential for proteins found in the PM, because digestive proteases traverse the PM in order to pass through to the gut lumen [46]. Disulfide bonds in SsPTP1 presumably have a comparable function. Indeed our experiments pointed towards substantial binding of the expressed CBD to chitin. Western blotting of chitin binding chromatography fractions confirmed that large amounts of TSP-CBD1 remained on the chitin matrix after thorough removal attempts using several elution buffers. In comparison, elution profiles of the positive control WGA showed the strongest binding. The weaker binding of the peritrophin fusion proteins could be due to i) the presence of only one instead of four chitin binding domains, ii) interference of the fused TSP protein, or iii) incorrect folding of the CBD domain. A recombinant peritrophin-like protein from *Fenneropenaeus chinensis* containing three CBDs revealed binding to a chitin matrix under similar conditions, but could be completely removed from the beads by SDS elution [47]. In contrast, peritrophin-like proteins containing one or several CBDs from adult cat flea (*Ctenocephalides felis*) did not show chitin binding activity suggesting additional functions of these domains, because adult cat fleas do not produce a PM [48].

The PM in the gut of insects is made up of proteoglycans, proteins and chitin [27], [49]. It plays a role as a discriminating semi-permeable molecular matrix [50], and often separates the contents in the lumen from the epithelial cells forming the midgut lining [27], [28], [50]. The PM of some insects described so far can form a sock-like structure to contain the ingested meal [28], [51], [52]. At light microscopy magnification and under the fixation conditions described the scabies mite peritrophin SsPTP1 was found to be localized in the entire gut lumen (Fig. 4A). This was repeatedly observed in many individual mites and representative examples are shown. While no cross-reactions with other scabies mite proteins were seen by western analysis (Fig. 5A), binding of the antisera to other putative peritrophins containing similar chitin binding domains may be possible. Very few studies have examined the scabies mite gut structure. The foregut, midgut and hindgut are directly connected with no obvious constriction joining the mid and hindguts [53], with the midgut lacking a cuticle. No visible peritrophic matrix lining the gut was described, although it was queried in these studies. One of the functions of the PM of insects is to act as a protective barrier against the invasion of parasites and microorganisms into the gut epithelium [54]. Different forms of PM varying in solubility may be produced at different life stages by the same insect [28] and there is considerable variation between organisms [27]. The apparent distribution of SsPTP1 throughout the entire gut lumen may indicate an extended function of this protein within the mite gut.

Given the millennia of co-evolution between parasites and host, many pathogens have evolved a range of elaborate counterstrategies to evade complement [55]. Among the many mechanisms observed the capture of complement initiators (such as immunoglobulins) and the depletion of complement components due to binding to secreted pathogen molecules have been described for bacteria, viruses, fungi and parasites [19]. Glycoproteins in herpes viruses have Fc receptor properties and can deplete antibody recognition and activation of the classical pathway [56]. There is increasing evidence that microorganisms developed incredible fine tuning of activation and inhibition. Viruses ‘voluntarily’ activate complement through surface glycoproteins to become opsonized and enter host cells through complement receptors. At the same time they keep complement activation in check by other mechanisms [19]. It may be possible that the scabies mite peritrophin targets MBL in the gut lumen, thereby depleting it and avoiding MAC formation on the gut epithelial cells, in addition to the inactivation of complement factors by the SMIPP-Ss [26]. MBL is an oligomeric molecule made up of eighteen 32 kDa polypeptides that form a hexameric structure [57]. It is a pattern recognition molecule specific for mannose, fucose and N-acetyl glucosamine (GlcNAc) [58]. This allows binding to sugar arrays on the surfaces of microorganisms and invertebrates [59] but not to most human glycoprotein glycans terminating in galactose or sialic acid. MBL thereby triggers the lectin pathway in the host serum to eliminate microbial and parasitic intruders [55]. The abundance of SsPTP1 within the mite gut and its high degree of predicted glycosylation led us to test the possible interaction between MBL and native SsPTP1 in extracts of native total mite protein.

Peritrophins vary greatly in molecular weight with the 12.5 kDa Ag-Aper1 of *A.gambiae* being the smallest and the 400 kDa invertebrate intestinal mucin of *Trichoplusia ni* being the largest known to date. On SDS-PAGE native SsPTP1 was detected in mite extracts as a single band running higher than expected. The binding of peritrophins within the PM to chitin and other proteins is unclear and some peritrophins may require strong denaturants to be solubilized [28]. The conditions used here in the preparation and the SDS-PAGE may not have completely dissociated SsPTP1 from other matrix components. In addition, the large number of negatively charged amino acids in the sequence may have reduced SDS-binding during electrophoresis, and the relatively high number of prolines may result in extended structures [46]. Repetitive antigens displaying large arrays of hydrophilicity have shown anomalous migration on SDS-PAGE [60], [61], [62]. Another obvious reason for the difference in experimental and theoretical molecular mass is glycosylation. The full extent of the glycosylation of SsPTP1 may not be addressed by our experiment as insoluble matrix structures may have interfered with the enzymatic digestion of the glycans. Carbohydrates can comprise up to 50% of the molecular mass of invertebrate intestinal PM associated mucins [38] with proline residues interspersed among the glycosylated residues contributing to a rigid conformation that may be important for resistance to host and pathogen proteases. Besides providing protection from proteolytic degradation, glycosylation is also thought to avoid infection through the gut wall, physical damage and dehydration by lubricating the epithelium [63], [64], [65]. SsPTP1 was predicted to be strongly glycosylated as the amino acid sequence outside of the CBDs was covered with putative N- and O-glycosylation sites. With an average concentration of only ∼1.2 µg/ml MBL is relatively scarce in human serum [66]. Nonetheless the experimental removal of glycans affected the binding of MBL drastically (Fig. 5B), indicating that MBL may have bound to SsPTP1-carbohydrates. In accordance with our finding, oligosaccharides attached to the native glycoprotein peritrophin 95 from *Lucilia cuprina* larvae have been found to play an essential role in raising parasite inhibitory activity in host sera [67] and seem to be an important constraint in vaccine development [68]. Furthermore, vaccination trials against *Ornithodoros erraticus* mid-gut secreted proteins have shown to induce lethal gut damage, which was thought to be mediated by the activation of the complement system [69].

Protection of gut epithelium from complement lysis by salivary proteins released into the bloodmeal of heamtophagous hemiptera *Triatoma brasiliensis* was recently demonstrated *in situ* [70]. Using a forced feeding procedure individual nymphs were exposed to concentrated active human complement, while the ingestion of salivatory complement inhibitors was simultaneously prevented. The midgut epithelia of these nymphs showed increased levels of MAC and cell lysis, evidenced by immunoflourescence and propidium iodide staining respectively. Control animals, that were either naturally fed, allowing access of salivary complement inhibitors to the gut, or received inactivated complement showed minimal MAC deposition and cell death [70]. In the study presented here we have used the same antibodies specific for the human SC5b-9 complex to detect any MAC formation in histological sections of scabies mite infested skin. Interestingly, the levels of MAC detection did not exceed background staining while the complement component C9 was strongly detectable in the mite gut. Evidently the combined anti-complement mechanisms present in the mite gut seem to inhibit MAC formation and thus may prevent complement mediated gut damage.

It is highly likely that, apart from SsPTP1, other mechanisms are leading to complement inhibition in the mite gut. Elucidating in depth the molecular mechanisms that are involved in restricting complement activation within the mite gut and in the infested epidermal tissue will be vital for developing novel strategies of therapeutic intervention against scabies and associated bacterial infections.
