# Correction: Scabies Mite Peritrophins Are Potential Targets of Human Host Innate Immunity

**DOI:** 10.1371/journal.pntd.0012329

**Published:** 2024-07-11

**Authors:** Angela Mika, Priscilla Goh, Deborah C. Holt, Dave J. Kemp, Katja Fischer

In Fig 2, parts A and B present lanes that originated on different gels and blots. The Coomassie stained SDS-PAGE gel and the accompanying western blot were both spliced to remove a lane. During figure re-arrangement lane 1 of the western blot in Fig 2B was erroneously duplicated and stretched to represent lanes 4–6, which are blank in the original underlying blot.

**Fig 2:**
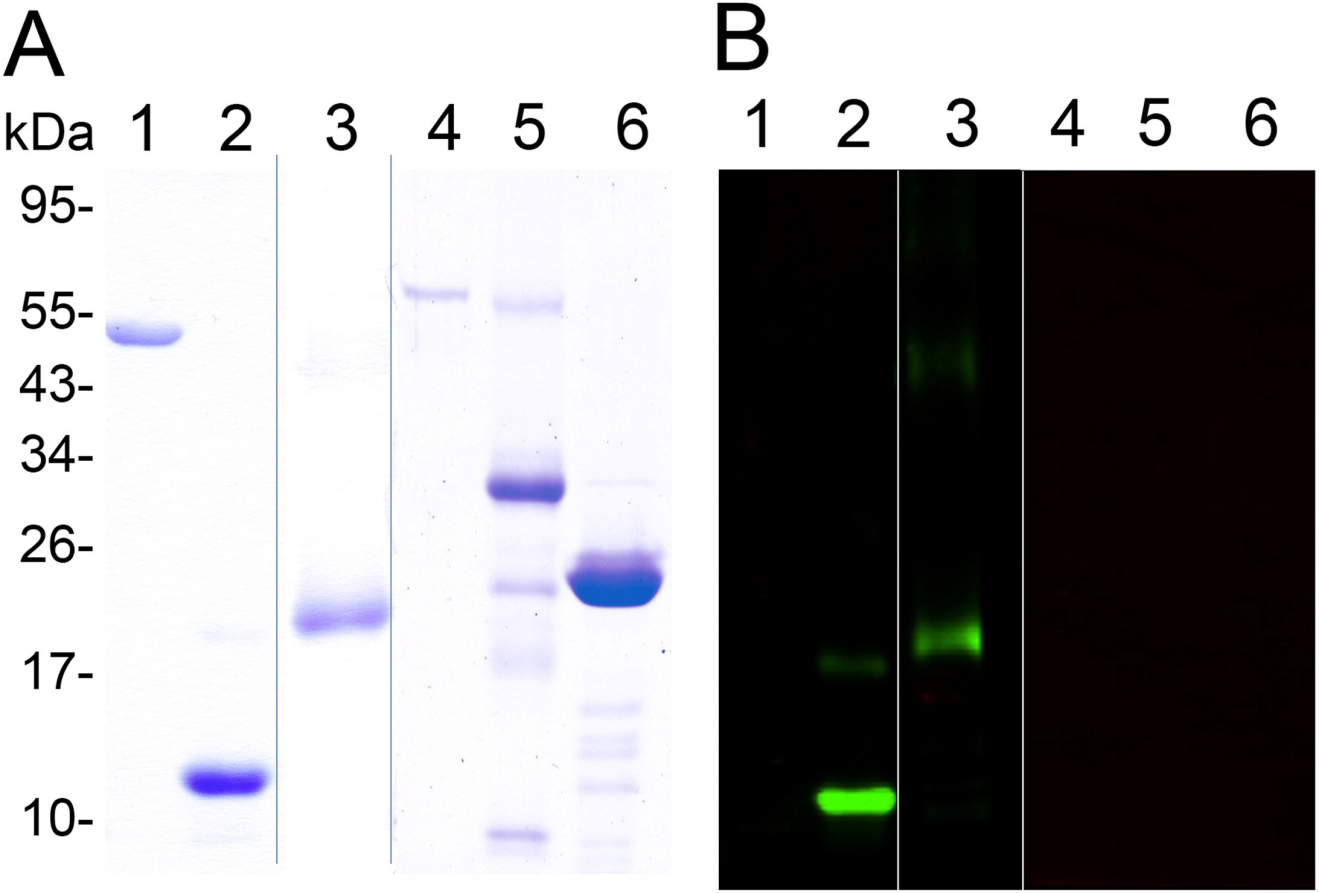
Specificity of mouse antisera raised against a recombinant chitin binding domain of SsPTP1. Shown is a series of purified scabies mite proteins on a coomassie-stained SDS-PAGE (A) and a corresponding western with a peritrophin-specific antibody (B). The gel (A) demonstrates the purity of the recombinant peptide CBD1 (lane 2) and the fusion protein TSP-CBD1 (lane 3) and (B) the specificity of the antibody raised against CBD1, confirming no cross-reaction with unrelated scabies mite proteins SMSB3a (lane 1), SMIPP-S D1 (lane 5), SMIPP-S I1 (lane 6) and BSA (lane 4). In (A) Lanes 1–3 and lanes 4–6 originate from separate gels that were re-arranged when a lane was removed. Blue lines indicate where the figure was re-arranged. In (B) Lanes 1–3 and lanes 4–6 originate from separate blots that were re-arranged when a lane was removed. White lines indicate where the figure was re-arranged. The underlying data for these figures are available in S1 File.

With this correction we provide an updated version of Fig 2 with lanes from different blots clearly indicated and notation of where lanes were re-arranged. The underlying uncropped gels and blots supporting this correction are provided in S1 File.

The original underlying data to support all results in the article and Supporting Information files are available from the corresponding author.

The authors apologize for the error in the published article.

## Supporting information

S1 File Underlying blots. (PPTX)
