# Supplementary figures and images for: Correction: Scabies Mite Peritrophins Are Potential Targets of Human Host Innate Immunity
Source: PLoS Negl Trop Dis. 2024 Jul 11;18(7):e0012329. doi: 10.1371/journal.pntd.0012329 (PMC11239068; doi:10.1371/journal.pntd.0012329)

## Slide 1
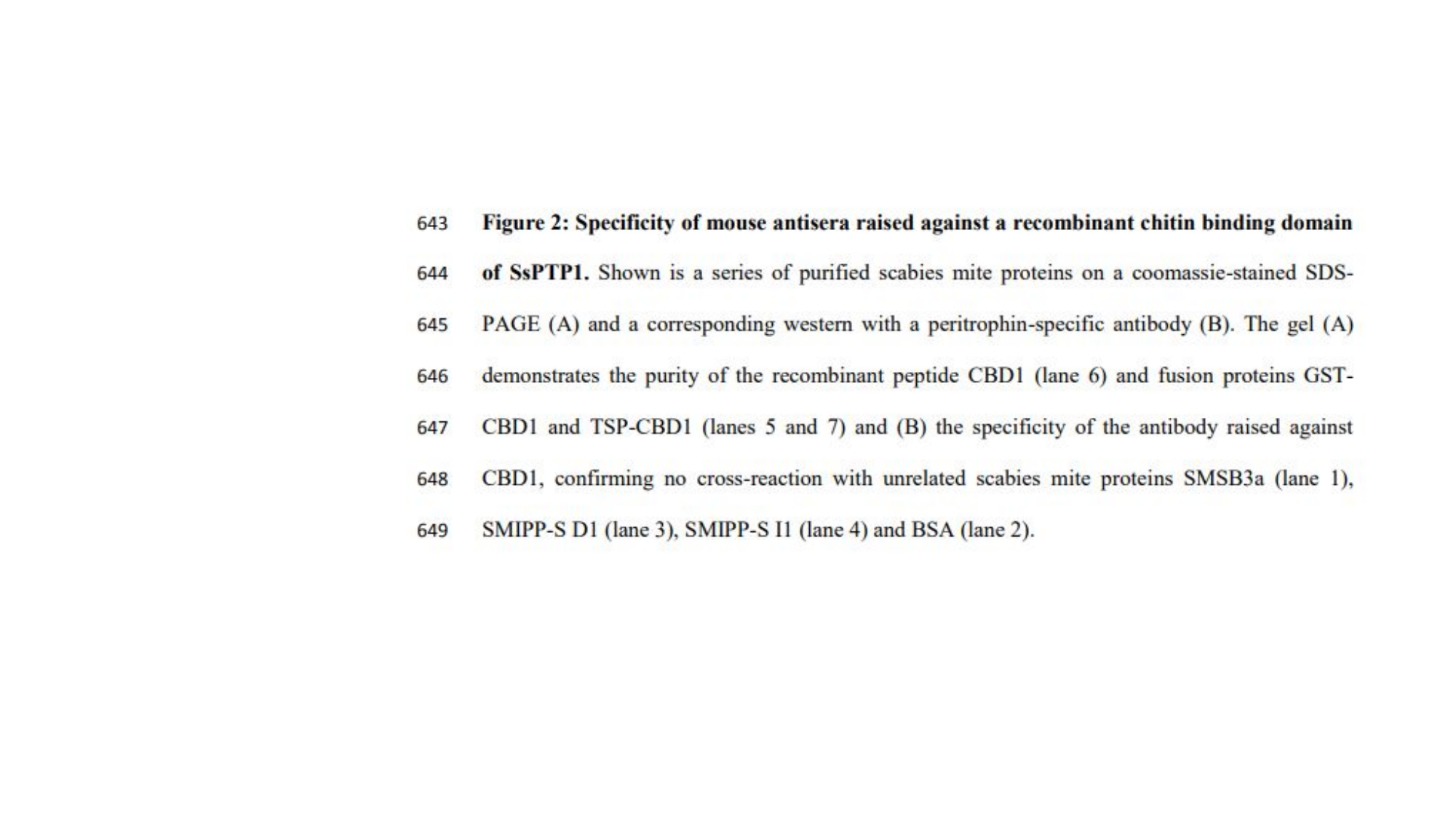

## Slide 2
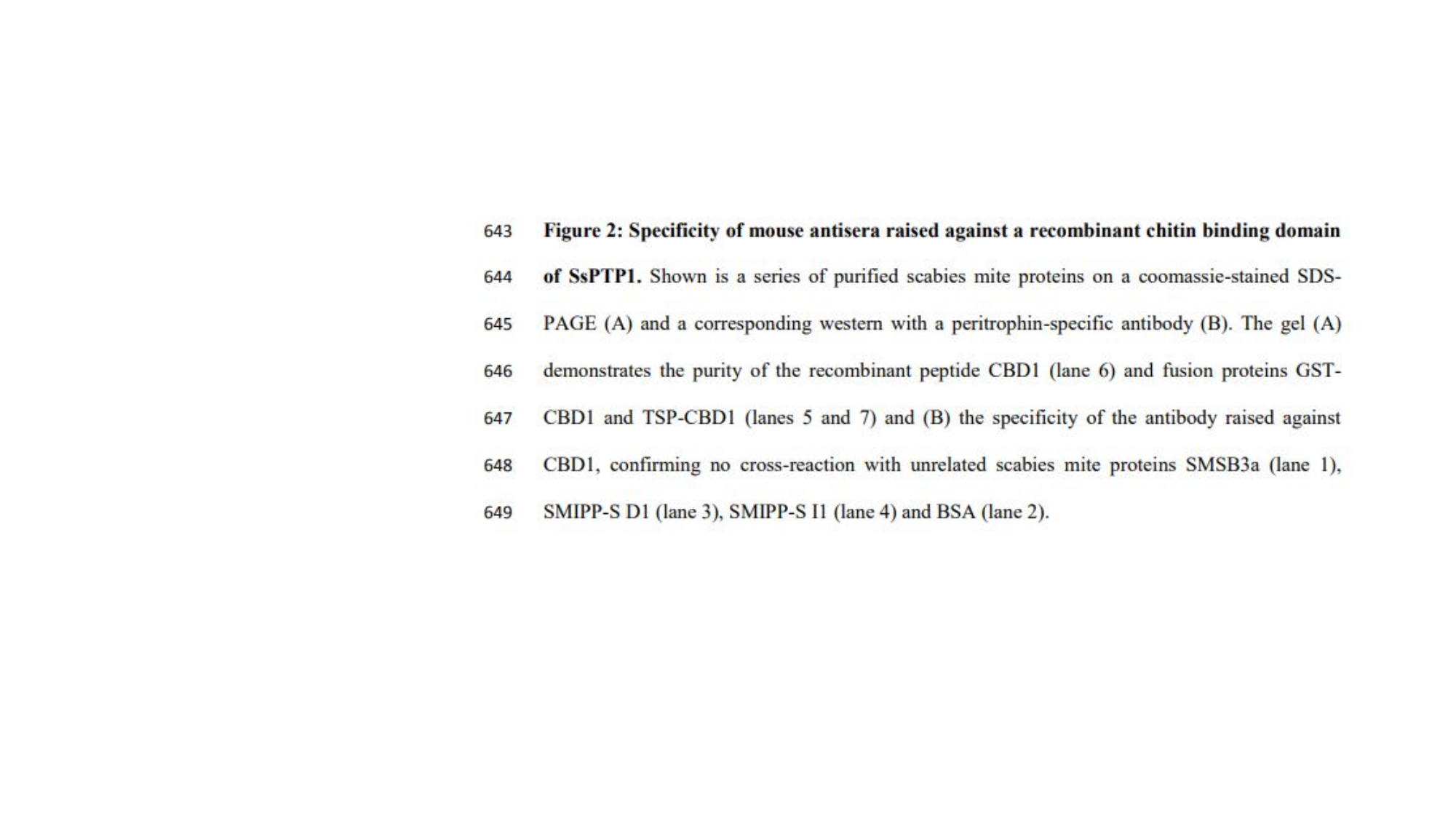

Supplement: S1 File — (PPTX) [file pntd.0012329.s001.pptx]
